# Supplementary figures and images for: The cure rate after different treatments for mucosal leishmaniasis in the Americas: A systematic review
Source: PLoS Negl Trop Dis. 2022 Nov 17;16(11):e0010931. doi: 10.1371/journal.pntd.0010931 (PMC9714886; doi:10.1371/journal.pntd.0010931)

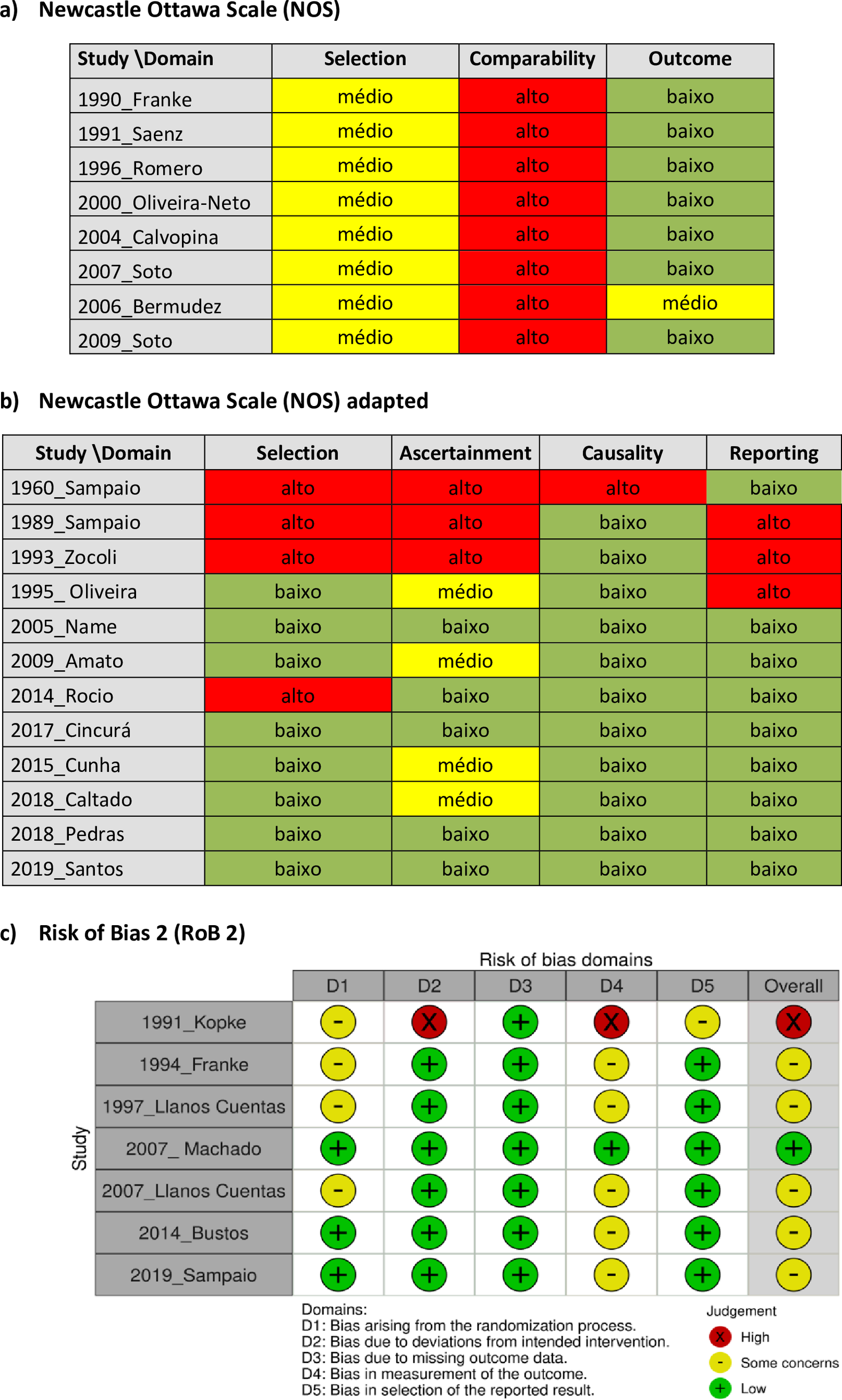

Supplement: S1 Fig — (TIF) [file pntd.0010931.s001.tif]
