# Supplementary material for: The cure rate after different treatments for mucosal leishmaniasis in the Americas: A systematic review
Source: PLoS Negl Trop Dis. 2022 Nov 17;16(11):e0010931. doi: 10.1371/journal.pntd.0010931 (PMC9714886; doi:10.1371/journal.pntd.0010931)
Supplement: S2 Table — (DOCX) [file pntd.0010931.s003.docx]

**S2 Table. Search strategies performed on December 15th 2021.**

| **Databases** | **Strategy used** | **Number of Studies** |
| --- | --- | --- |
| MEDLINE (Pubmed) | ((((("Leishmaniasis, Mucocutaneous"[MeSH Terms]) OR ("Leishmaniasis, Mucocutaneous"[Title/Abstract])) OR ("Leishmaniasis Mucocutaneous"[Title/Abstract])) OR ("Mucocutaneous Leishmaniasis"[Title/Abstract])) OR ("Mucocutaneous Leishmaniasis"[Title/Abstract])) AND ((((((((((((((((((((((((((("Antiprotozoal Agents"[Title/Abstract]) OR ("Amphotericin B"[Title/Abstract])) OR (fungizone[Title/Abstract])) OR ("Amphotericin B Cholesterol Dispersion"[Title/Abstract])) OR ("Amphotericin B Colloidal Dispersion"[Title/Abstract])) OR (amphocil[Title/Abstract])) OR (abelcet[Title/Abstract])) OR (ambisome[Title/Abstract])) OR ("Liposomal Amphotericin B"[Title/Abstract])) OR ("amphotericin B lipid complex"[Title/Abstract])) OR ("Meglumine Antimoniate"[Title/Abstract])) OR (meglumine[Title/Abstract])) OR (glucantime[Title/Abstract])) OR ("N-Methylglucamine Antimonate"[Title/Abstract])) OR ("Antimony Sodium Gluconate"[Title/Abstract])) OR (stibogluconate[Title/Abstract])) OR ("Sodium Stibogluconate"[Title/Abstract])) OR (pentostam[Title/Abstract])) OR (pentamidine[Title/Abstract])) OR (pentoxifylline[Title/Abstract])) OR (fluconazole[Title/Abstract])) OR (itraconazole[Title/Abstract])) OR (miltefosine[Title/Abstract])) OR (paramomycin[Title/Abstract])) OR (allopurinol[Title/Abstract])) OR (therapy[Title/Abstract])) OR (efficacy[Title/Abstract])) | 323 |
| EMBASE | ('leishmaniasis mucocutaneous' OR 'tegumentary leishmaniasis'/exp OR 'tegumentary leishmaniasis' OR 'new world leishmaniasis'/exp OR 'new world leishmaniasis') AND ('therapy'/exp OR 'combination therapy':ti,ab,kw OR 'disease therapy':ti,ab,kw OR 'disease treatment':ti,ab,kw OR 'diseases treatment':ti,ab,kw OR 'disorder treatment':ti,ab,kw OR 'disorders treatment':ti,ab,kw OR 'efficacy, therapeutic':ti,ab,kw OR 'illness treatment':ti,ab,kw OR 'medical therapy':ti,ab,kw OR 'medical treatment':ti,ab,kw OR 'multiple therapy':ti,ab,kw OR 'polytherapy':ti,ab,kw OR 'somatotherapy':ti,ab,kw OR 'therapeutic action':ti,ab,kw OR 'therapeutic efficacy':ti,ab,kw OR 'therapeutic trial':ti,ab,kw OR 'therapeutic trials':ti,ab,kw OR 'therapeutics':ti,ab,kw OR 'therapy':ti,ab,kw OR 'therapy, medical':ti,ab,kw OR 'treatment effectiveness':ti,ab,kw OR 'treatment efficacy':ti,ab,kw OR 'treatment, medical':ti,ab,kw OR 'amphotericin b'/mj OR 'antimony'/mj OR 'pentoxifylline'/mj OR 'miltefosine'/mj OR 'itraconazole'/mj OR 'fluconazole'/mj OR 'meglumine'/mj OR 'meglumine antimonate'/exp/mj OR 'allopurinol'/exp OR 'pentostam':ti,ab,kw OR 'sodium stibogluconate':ti,ab,kw OR 'solustibosan':ti,ab,kw OR 'solustin':ti,ab,kw OR 'solusurmin':ti,ab,kw OR 'solyusurmin':ti,ab,kw OR 'stibanate':ti,ab,kw OR 'stibanose':ti,ab,kw OR 'stibatin':ti,ab,kw OR 'stibinol':ti,ab,kw OR 'stibogluconate sodium':ti,ab,kw OR 'stibonate':ti,ab,kw OR 'wr 229870al':ti,ab,kw OR efficac:ti,ab) | 350 |
| BVS (LILACS) | ((mh:("Leishmaniose Mucocutânea")) OR (mh:("Leishmaniasis, Mucocutaneous")) OR (mh:("Leishmaniasis Mucocutánea")) OR ("Leishmaniose Mucocutânea") OR ("Leishmaniasis, Mucocutaneous") OR ("Leishmaniasis Mucocutánea") OR ("Leishmaniasis Mucocutaneous") OR ("Mucocutaneous Leishmaniasis") OR ("Mucocutaneous Leishmaniasis") OR ("Mucocutânea Leishmaniose") OR ("Mucocutánea Leishmaniasis") ) AND (("Antiprotozoários") OR ("Antiprotozoal Agents") OR ("Antiprotozoarios") OR ("Anfotericina B") OR ("Amphotericin B") OR (fungizone) OR ("Dispersão de colesterol de anfotericina B") OR ("Amphotericin B Cholesterol Dispersion") OR ("Dispersão coloidal de anfotericina B") OR ("Amphotericin B Colloidal Dispersion") OR (amphocil) OR (abelcet) OR (ambisome) OR ("Anfotericina B Lipossomal") OR ("Liposomal Amphotericin B") OR ("Anfotericina B Complexo Lipídico") OR ("amphotericin B lipid complex") OR ("Antimoniato de Meglumina") OR ("Meglumine Antimoniate") OR (meglumina) OR (meglumine) OR (Glucantime) OR ("Antimoniato de N-Metilglucamina") OR ("N-Methylglucamine Antimonate") OR ("Gluconato de Antimônio e Sódio") OR ("Antimony Sodium Gluconate") OR ("Gluconato de Sodio Antimonio") OR (Estibogluconato) OR (Stibogluconate) OR ("Estibogluconato de Sódio") OR ("Sodium Stibogluconate") OR (pentostam) OR (pentamidina) OR (pentamidine) OR (pentoxifilina) OR (pentoxifylline) OR (fluconazol) OR (fluconazole) OR (itraconazol) OR (itraconazole) OR (miltefosina) OR (miltefosine) OR (Paromomicina) OR (Paromomycin) OR (Alopurinol) OR (Allopurinol) OR (Terapia) OR (therapy) OR (Terapêutica) OR (Therapeutics) OR (Terapéutica) OR (Eficácia) OR (efficacy) OR (Eficacia)) | 200 |
| Web of Science | ((ALL=("Leishmaniasis, Mucocutaneous")) OR ALL=("Leishmaniasis Mucocutaneous")) OR ALL=("Mucocutaneous Leishmaniasis") AND ((((((((((((((((((((((((((ALL=("Antiprotozoal Agents")) OR ALL=("Amphotericin B")) OR ALL=(fungizone)) OR ALL=("Amphotericin B Cholesterol Dispersion")) OR ALL=("Amphotericin B Colloidal Dispersion")) OR ALL=(amphocil)) OR ALL=(abelcet)) OR ALL=(ambisome)) OR ALL=("Liposomal Amphotericin B")) OR ALL=("amphotericin B lipid complex")) OR ALL=("Meglumine Antimoniate")) OR ALL=(meglumine)) OR ALL=(glucantime)) OR ALL=("N-Methylglucamine Antimonate")) OR ALL=("Antimony Sodium Gluconate")) OR ALL=(stibogluconate)) OR ALL=("Sodium Stibogluconate")) OR ALL=(pentostam)) OR ALL=(pentamidine)) OR ALL=(pentoxifylline)) OR ALL=(fluconazole)) OR ALL=(itraconazole)) OR ALL=(miltefosine)) OR ALL=(paramomycin)) OR ALL=(allopurinol)) OR ALL=(therapy)) OR ALL=(efficacy) | 231 |
