# Supplementary material for: The cure rate after different treatments for mucosal leishmaniasis in the Americas: A systematic review
Source: PLoS Negl Trop Dis. 2022 Nov 17;16(11):e0010931. doi: 10.1371/journal.pntd.0010931 (PMC9714886; doi:10.1371/journal.pntd.0010931)
Supplement: S3 Table — &: AE were considered per treatment and not per patient. *the number of participants reporting AE is different from the number of participants reporting a cure rate. ABLC: Amphotericin B, lipid complex. AE: Adverse Event. AS: Aminosidine sulphate. c-AMB: Amphotericin B colloidal dispersion. CTCAE: Common Terminology Criteria for Adverse Events. DAIDS: Division of AIDS Table for Grading of Severity of Adult and Pediatric Adverse Events. d-AMB: Deoxycholate amphotericin B. HLGT: High Level Group Terms. L-AMB: Liposomal amphotericin B. MA: Meglumine antimonate. MA-LD: Meglumine antimonate low dose. MF: Miltefosine. NEC: not elsewhere classified. NR: not reported. PENT: pentamidine. PT: Preferred Term. Sbv: Antimonial pentavalent. SOC: System Organ Classes. SSG: Sodium stibogluconate. (DOCX) [file pntd.0010931.s004.docx]

**S3 table. Adverse Events according to therapy arm in each study**

| **Year, Author** | **Therapy (number of treated patients)** | **PT of Adverse event** | **HLGT of Adverse event** | **SOC of Adverse event** | **Number of AE reported** | **AE adopted classification** | **Number of patients with severe AE or with treatment suspended due to AE** | **Summary of description by the authors** |
| --- | --- | --- | --- | --- | --- | --- | --- | --- |
| 2019, Sampaio | MA (18) | Electrocardiogram QT prolonged | Cardiac and vascular investigations (excl enzyme tests) | Investigations | 2 | Treatment suspended due to AE | 2 | In the MF group one patient had the treatment suspended due to abdominal pain and elevation of serum amylase. In the MA group, two patients had the treatment suspended due to prolonged corrected QTc interval. |
|  | MF (20) | Abdominal pain | Gastrointestinal signs and symptoms | Gastrointestinal disorders | 1 |  | 1 |  |
|  |  | Amylase increased | Gastrointestinal investigations | Investigations | 1 |  |  |  |
| 2019, Santos **^&^** | ABLC (13) | Infusion related reaction | Procedural related injuries and complications NEC | Injury, poisoning and procedural complications | 10 | NR |  | Approximately 66% of the patients presented with AEs during ML treatment. The most frequent symptoms were infusion-related AEs (fever, chills, sweating, and palpitations) in 48.1% of the participants, followed by phlebitis (20.2%) and nausea/vomiting (18.3%). The most common systemic AEs were electrolyte imbalance (28.8%) and acute kidney injury (19.2%). d-AMB had worse AEs than other regimens. ABLC was associated with infusion-related AEs. Meanwhile, antimonial pentavalent was associated with metabolic disturbances, such as hyperamylasemia and increased liver enzymes. However, fewer electrolyte disorders were observed when amphotericin is used. |
|  |  | Phlebitis | Vascular infections and inflammations | Vascular disorders | 6 |  |  |  |
|  |  | Vomiting | Gastrointestinal signs and symptoms | Gastrointestinal disorders | 5 |  |  |  |
|  |  | Fever | Body temperature conditions | General disorders and administration site conditions | 3 |  |  |  |
|  |  | Headache | Headaches | Nervous system disorders | 1 |  |  |  |
|  |  | Chest pain | General system disorders NEC | General disorders and administration site conditions | 3 |  |  |  |
|  |  | Tremor | Movement disorders (incl parkinsonism) | Nervous system disorders | 4 |  |  |  |
|  |  | Chills | General system disorders NEC | General disorders and administration site conditions | 2 |  |  |  |
|  |  | Muscle pain | Muscle disorders | Musculoskeletal and connective tissue disorders | 1 |  |  |  |
|  |  | Lumbar pain | Musculoskeletal and connective tissue disorders NEC | Musculoskeletal and connective tissue disorders | 1 |  |  |  |
|  |  | Palpitations | Cardiac disorders, signs and symptoms NEC | Cardiac disorders | 1 |  |  |  |
|  |  | Hyperhidrosis | General system disorders NEC | General disorders and administration site conditions | 2 |  |  |  |
|  |  | Electrolyte imbalance | Electrolyte and fluid balance conditions | Metabolism and nutrition disorders | 6 |  |  |  |
|  |  | Acute kidney injury | Renal disorders (excl nephropathies) | Renal and urinary disorders | 1 |  |  |  |
|  |  | Myelopathy | Spinal cord and nerve root disorders | Nervous system disorders | 1 |  |  |  |
|  | d-AMB (14) | Infusion related reaction | Procedural related injuries and complications NEC | Injury, poisoning and procedural complications | 7 |  |  |  |
|  |  | Phlebitis | Vascular infections and inflammations | Vascular disorders | 3 |  |  |  |
|  |  | Vomiting | Gastrointestinal signs and symptoms | Gastrointestinal disorders | 4 |  |  |  |
|  |  | Fever | Body temperature conditions | General disorders and administration site conditions | 1 |  |  |  |
|  |  | Headache | Headaches | Nervous system disorders | 3 |  |  |  |
|  |  | Chills | General system disorders NEC | General disorders and administration site conditions | 3 |  |  |  |
|  |  | Electrolyte imbalance | Electrolyte and fluid balance conditions | Metabolism and nutrition disorders | 7 |  |  |  |
|  |  | Acute kidney injury | Renal disorders (excl nephropathies) | Renal and urinary disorders | 8 |  |  |  |
|  | L-AMB (32) | Infusion related reaction | Procedural related injuries and complications NEC | Injury, poisoning and procedural complications | 17 |  |  |  |
|  |  | Phlebitis | Vascular infections and inflammations | Vascular disorders | 6 |  |  |  |
|  |  | Vomiting | Gastrointestinal signs and symptoms | Gastrointestinal disorders | 4 |  |  |  |
|  |  | Fever | Body temperature conditions | General disorders and administration site conditions | 3 |  |  |  |
|  |  | Headache | Headaches | Nervous system disorders | 2 |  |  |  |
|  |  | Chest pain | General system disorders NEC | General disorders and administration site conditions | 4 |  |  |  |
|  |  | Tremor | Movement disorders (incl parkinsonism) | Nervous system disorders | 3 |  |  |  |
|  |  | Chills | General system disorders NEC | General disorders and administration site conditions | 1 |  |  |  |
|  |  | Muscle pain | Muscle disorders | Musculoskeletal and connective tissue disorders | 2 |  |  |  |
|  |  | Lumbar pain | Musculoskeletal and connective tissue disorders NEC | Musculoskeletal and connective tissue disorders | 4 |  |  |  |
|  |  | Palpitations | Cardiac disorders, signs and symptoms NEC | Cardiac disorders | 3 |  |  |  |
|  |  | Hyperhidrosis | General system disorders NEC | General disorders and administration site conditions | 1 |  |  |  |
|  |  | Electrolyte imbalance | Electrolyte and fluid balance conditions | Metabolism and nutrition disorders | 12 |  |  |  |
|  |  | Acute kidney injury | Renal disorders (excl nephropathies) | Renal and urinary disorders | 11 |  |  |  |
|  | Itraconazole (10) | - | - | - | 0 |  |  |  |
|  | Pentamidine (11) | Infusion related reaction | Procedural related injuries and complications NEC | Injury, poisoning and procedural complications | 5 |  |  |  |
|  |  | Phlebitis | Vascular infections and inflammations | Vascular disorders | 4 |  |  |  |
|  |  | Vomiting | Gastrointestinal signs and symptoms | Gastrointestinal disorders | 3 |  |  |  |
|  |  | Headache | Headaches | Nervous system disorders | 1 |  |  |  |
|  |  | Malaise | General system disorders NEC | General disorders and administration site conditions | 1 |  |  |  |
|  |  | Electrolyte imbalance | Electrolyte and fluid balance conditions | Metabolism and nutrition disorders | 4 |  |  |  |
|  |  | Myelopathy | Spinal cord and nerve root disorders | Nervous system disorders | 1 |  |  |  |
|  |  | Hypoglycaemia | Glucose metabolism disorders (incl diabetes mellitus) | Metabolism and nutrition disorders | 1 |  |  |  |
|  | Sb^v^ (25) | Infusion related reaction | Procedural related injuries and complications NEC | Injury, poisoning and procedural complications | 11 |  |  |  |
|  |  | Phlebitis | Vascular infections and inflammations | Vascular disorders | 2 |  |  |  |
|  |  | Vomiting | Gastrointestinal signs and symptoms | Gastrointestinal disorders | 3 |  |  |  |
|  |  | Fever | Body temperature conditions | General disorders and administration site conditions | 2 |  |  |  |
|  |  | Headache | Headaches | Nervous system disorders | 2 |  |  |  |
|  |  | Chest pain | General system disorders NEC | General disorders and administration site conditions | 1 |  |  |  |
|  |  | Muscle pain | Muscle disorders | Musculoskeletal and connective tissue disorders | 3 |  |  |  |
|  |  | Palpitations | Cardiac disorders, signs and symptoms NEC | Cardiac disorders | 1 |  |  |  |
|  |  | Arthralgia | Joint disorders | Musculoskeletal and connective tissue disorders | 4 |  |  |  |
|  |  | Gastrointestinal disorders | Gastrointestinal conditions NEC | Gastrointestinal disorders | 3 |  |  |  |
|  |  | Malaise | General system disorders NEC | General disorders and administration site conditions | 1 |  |  |  |
|  |  | Rash | Epidermal and dermal conditions | Skin and subcutaneous tissue disorders | 1 |  |  |  |
|  |  | Electrolyte imbalance | Electrolyte and fluid balance conditions | Metabolism and nutrition disorders | 1 |  |  |  |
|  |  | Hepatic enzyme abnormal | Hepatobiliary investigations | Investigations | 3 |  |  |  |
|  |  | Hyperamylasemia | Metabolism disorders NEC | Metabolism and nutrition disorders | 7 |  |  |  |
|  |  | Electrocardiogram abnormal | Cardiac and vascular investigations (excl enzyme tests) | Investigations | 5 |  |  |  |
|  |  | Myelopathy | Spinal cord and nerve root disorders | Nervous system disorders | 1 |  |  |  |
| 2018, Cataldo | MA-LD (27) | NR | NR | NR | 19 | Toxicity scale adapted from Division of AIDS Table for Grading of Severity of Adult and Pediatric Adverse Events (DAIDS) | 2 (laboratory events in ML/RJ group) | Regarding ML patients, the occurrence of adverse events was 58% in the RJ group (7/12) and 80% in the OS group (12/15). Permanent discontinuation of treatment due to adverse events was not required for any patient.  The author presents a figure with classification by severity (separating ML and CL) and a table with AE per patient (without separation by clinical form). For ML, 2 laboratory events were classified as severe. |
| 2018, Pedras | Fluconazole (9) | Nausea | Gastrointestinal signs and symptoms | Gastrointestinal disorders | 1 | NR |  | Was observed that the adverse events rate is not negligible, reinforcing caution in using this approach. |
|  |  | Hyporexia | Appetite and general nutritional disorders | Metabolism and nutrition disorders | 1 |  |  |  |
|  |  | Hepatic enzyme increased | Hepatobiliary investigations | Investigations | 1 |  |  |  |
|  | L-AMB (9) | Abdominal pain | Gastrointestinal signs and symptoms | Gastrointestinal disorders | 1 | NR |  | In recent years, lipid formulations of amphotericin B have been considered as the most attractive treatment modalities for ML, due to better safety profile. |
|  |  | Nausea | Gastrointestinal signs and symptoms | Gastrointestinal disorders | 1 |  |  |  |
|  |  | Hepatic enzyme increased | Hepatobiliary investigations | Investigations | 1 |  |  |  |
|  |  | Blood creatinine increased | Renal and urinary tract investigations and urinalyses | Investigations | 2 |  |  |  |
|  | MA (17) | Abdominal pain | Gastrointestinal signs and symptoms | Gastrointestinal disorders | 1 | NR |  | The high frequency of adverse events during antimonial therapy is clearly demonstrated in the table presented by the author. |
|  |  | Diarrhoea | Gastrointestinal motility and defaecation conditions | Gastrointestinal disorders | 1 |  |  |  |
|  |  | Nausea | Gastrointestinal signs and symptoms | Gastrointestinal disorders | 4 |  |  |  |
|  |  | Vomiting | Gastrointestinal signs and symptoms | Gastrointestinal disorders | 2 |  |  |  |
|  |  | Myalgia | Muscle disorders | Musculoskeletal and connective tissue disorders | 13 |  |  |  |
|  |  | Arthralgia | Joint disorders | Musculoskeletal and connective tissue disorders | 9 |  |  |  |
|  |  | Hyporexia | Appetite and general nutritional disorders | Metabolism and nutrition disorders | 3 |  |  |  |
|  |  | Dizziness | Neurological disorders NEC | Nervous system disorders | 1 |  |  |  |
|  |  | Fever | Body temperature conditions | General disorders and administration site conditions | 1 |  |  |  |
|  |  | Rash | Epidermal and dermal conditions | Skin and subcutaneous tissue disorders | 1 |  |  |  |
|  |  | Hepatic enzyme increased | Hepatobiliary investigations | Investigations | 5 |  |  |  |
|  |  | Blood creatinine increased | Renal and urinary tract investigations and urinalyses | Investigations | 1 |  |  |  |
|  |  | Lipase increased | Gastrointestinal investigations | Investigations | 9 |  |  |  |
|  |  | Electrocardiogram abnormal | Cardiac and vascular investigations (excl enzyme tests) | Investigations | 6 |  |  |  |
| 2017, Cincurá | MA or  MA + pentoxifyline (251) | NR | NR | NR | NR | NR |  | NR |
| 2015, Cunha | L-AMB (29) | Renal failure | Renal disorders | Renal and urinary disorders | 5 | NR |  | Seven patients presented with adverse effects during the treatment: five patients (17.2%) reported kidney failure, there was one report of myalgia and one of fever, both during the infusion. |
|  |  | Myalgia | Muscle disorders | Musculoskeletal and connective tissue disorders | 1 |  |  |  |
|  |  | Fever | Body temperature conditions | General disorders and administration site conditions | 1 |  |  |  |
| 2014, Bustos | MA (10) | Asthenia | General system disorders NEC | General disorders and administration site conditions | 3 | Common Terminology Criteria for Adverse Events (CTCAE) |  | AE occurred in 6/10 patients, mostly during the first week of treatment, of variable duration throughout the period of administration of therapy, and required administration of anti-inflammatory drugs. The highest incidence (3/10) was for asthenia, arthralgia (localized or generalized), and headache. Other effects occurred in 2/10 patients and included pain at the injection site, fever, and myalgia (localized or generalized). One patient presented with a self-limited generalized skin rash, lasting 24 hours, during the first week of therapy administration and another patient presented electrocardiographic changes (QT prolongation) during the last week of treatment (week 4), which reverted to control after 15 days from the end of treatment. |
|  |  | Arthralgia | Joint disorders | Musculoskeletal and connective tissue disorders | 3 |  |  |  |
|  |  | Headache | Headaches | Nervous system disorders | 3 |  |  |  |
|  |  | Injection site pain | Administration site reactions | General disorders and administration site conditions | 2 |  |  |  |
|  |  | Fever | Body temperature conditions | General disorders and administration site conditions | 2 |  |  |  |
|  |  | Myalgia | Muscle disorders | Musculoskeletal and connective tissue disorders | 2 |  |  |  |
|  |  | Rash | Epidermal and dermal conditions | Skin and subcutaneous tissue disorders | 1 |  |  |  |
|  |  | Electrocardiogram QT prolonged | Cardiac and vascular investigations (excl enzyme tests) | Investigations | 1 |  |  |  |
|  | MF (9) | Gastrointestinal disorders | Gastrointestinal conditions NEC | Gastrointestinal disorders | 8 |  |  | AE occurred in 8/9 patients, with the highest incidence of gastrointestinal disorders (8/9). These corresponded to dyspeptic symptoms, of which the most frequent was vomiting (5/9); nausea, epigastric pain, epigastric distention, early satiety, and postprandial fullness occurred in 2/9 patients. In all cases, the severity of the gastrointestinal AE was grade 1 (mild symptoms). One patient also presented increased uremia and amylasemia (less than 2 times its normal value) at the end of the first week of treatment, which normalized at the end of the second week of therapy administration. |
|  |  | Azotaemia | Renal disorders (excl nephropathies) | Renal and urinary disorders | 1 |  |  |  |
|  |  | Amylase decreased | Gastrointestinal investigations | Investigations | 1 |  |  |  |
| 2014, Rocio | L-AMB (16) | Infusion related reaction | Procedural related injuries and complications NEC | Injury, poisoning and procedural complications | 14 | Treatment suspended due to AE | 5 (stop the drug due to acute renal failure) | Fourteen patients had adverse effects using liposomal amphotericin B (infusionrelated: chills, fever, phlebitis, nausea/vomiting, headache, chest pain, back pain and palpitation; systemic: electrolyte disorder and acute kidney injury). Of these patients, five had to stop the drug due to acute renal failure, but two of them restarted liposomal amphotericin B after improvement of renal function and were considered cured at the follow-up. |
| 2009, Amato | c-AMB (9) d-AMB (17) L-AMB (4) Intraconazole (15) MA (73) PENT (22) | NR |  |  | NR | NR |  | NR |
| 2009, Soto | MF (42)* | Gastrointestinal disorders | Gastrointestinal conditions NEC | Gastrointestinal disorders | 31 | Common Terminology Criteria for Adverse Events (CTCAE) |  | In our analysis, we considered only group C. However, the author describes that 31 of 42 patients (group B + group C) reported gastrointestinal symptoms of common toxicity criteria grade 1 severity 1 (CTCAE) (up to 1 vomiting episode, 2 to 3 episodes of excess stools, mild anorexia, nausea, or abdominal pain), lasting an average of 4 days (range = 1-10 days). |
| 2007, Llanos-Cuentas | MA (17) | Fever | Body temperature conditions | General disorders and administration site conditions | 4 | NR |  | There were no serious adverse events with either drug, nor any differences in the rates of hematologic and serum chemistry abnormalities. Transient and mild EKG abnormalities were observed in the MA group that did not need therapeutic intervention. AS was associated with pain at the injection site that improved with the application of local heat. |
|  |  | Chills | General system disorders NEC | General disorders and administration site conditions | 7 |  |  |  |
|  |  | Arthralgia | Joint disorders | Musculoskeletal and connective tissue disorders | 11 |  |  |  |
|  |  | Decreased appetite / anorexia | Appetite and general nutritional disorders | Metabolism and nutrition disorders | 4 |  |  |  |
|  |  | Myalgia | Muscle disorders | Musculoskeletal and connective tissue disorders | 11 |  |  |  |
|  | AS (21) | Fever | Body temperature conditions | General disorders and administration site conditions | 3 |  |  |  |
|  |  | Chills | General system disorders NEC | General disorders and administration site conditions | 5 |  |  |  |
|  |  | Arthralgia | Joint disorders | Musculoskeletal and connective tissue disorders | 9 |  |  |  |
|  |  | Decreased appetite / anorexia | Appetite and general nutritional disorders | Metabolism and nutrition disorders | 1 |  |  |  |
|  |  | Myalgia | Muscle disorders | Musculoskeletal and connective tissue disorders | 8 |  |  |  |
| 2007, Machado | MA (12) | Decreased appetite / anorexia | Appetite and general nutritional disorders | Metabolism and nutrition disorders | 1 | Treatment suspended due to AE |  | Mild adverse effects were observed more frequently in the MA + pentoxifyline group, including nausea (3 patients); arthralgias (1 patient); and dizziness, abdominal pain, and diarrhea (1 patient).  In the group treated with MA, 1 patient complained of anorexia, nausea, and myalgias. No patients in either group discontinued treatment because of these adverse effects. |
|  |  | Nausea | Gastrointestinal signs and symptoms | Gastrointestinal disorders | 1 |  |  |  |
|  |  | Myalgia | Muscle disorders | Musculoskeletal and connective tissue disorders | 1 |  |  |  |
|  | MA + pentoxifyline (11) | Nausea | Gastrointestinal signs and symptoms | Gastrointestinal disorders | 3 |  |  |  |
|  |  | Arthralgia | Joint disorders | Musculoskeletal and connective tissue disorders | 1 |  |  |  |
|  |  | Dizziness | Neurological disorders NEC | Nervous system disorders | 1 |  |  |  |
|  |  | Abdominal pain | Gastrointestinal signs and symptoms | Gastrointestinal disorders | 1 |  |  |  |
|  |  | Diarrhoea | Gastrointestinal motility and defaecation conditions | Gastrointestinal disorders | 1 |  |  |  |
| 2007, Soto | d-AMB (19) | Vomiting | Gastrointestinal signs and symptoms | Gastrointestinal disorders | NR | Common Terminology Criteria for Adverse Events (CTCAE) | 3 | 3 patients discontinued treatment prematurely after 9, 17, and 18 injections because of adverse events (vomiting and/or increased creatinine levels and/or anaphylaxis. |
|  |  | Blood creatinine increased | Renal and urinary tract investigations and urinalyses | Investigations | NR |  |  |  |
|  | MF (78) | Nausea | Gastrointestinal signs and symptoms | Gastrointestinal disorders | NR |  |  | Miltefosine was well tolerated. Nausea, vomiting, and diarrhea were each reported by 8-17 patients, and episodes of nausea or vomiting were graded using the Common Toxicity Criteria. Mean values of liver function tests and kidney function tests did not change; a few patients had values that were slightly above the upper limit of normal after treatment. |
|  |  | Vomiting | Gastrointestinal signs and symptoms | Gastrointestinal disorders | NR |  |  |  |
|  |  | Diarrhoea | Gastrointestinal motility and defaecation conditions | Gastrointestinal disorders | NR |  |  |  |
| 2006, Bermudez | SSG (146)* | Arthralgia / Myalgia | Joint disorders / Muscle disorders | Musculoskeletal and connective tissue disorders | 12 | Adverse effects were only graded ‘severe’ if they interfered with the patient’s daily activities and/or required a temporary or permanent interruption of treatment. The difference between ‘mild’ and ‘moderate’ effects was based on the clinician’s subjective assessment. |  | Not reported separately for ML.  Forty-one (28.1%) of the 146 patients (CL + ML) developed at least one adverse effect: arthralgias and/or myalgias (8%), headache (7%), phlebitis (4%), pain at injection site (3%), malaise (3%), insomnia (3%), bradycardia (2%), fever (2%), vertigo (1%), weight loss (1%), nausea (1%), vomiting (1%), pruritus (1%) or anorexia (1%). Ten (6.8%) of the patients were considered to have had moderate adverse effect(s) but no fatalities or other severe adverse effects occurred. Compared with the CL patients, the ML patients were significantly more likely to have presented with one or more adverse effects (P=0.016), with an odds ratio and [95% confidence interval (CI)] of 2.8 [1.2–6.6]. A higher total dose of SSG (454 v. 426 mg/kg; P=0.078) and a larger induration in the leishmania skin test (13.1 v. 10.5 mm; P=0.019) were also associated with increased risk of adverse effect(s), whereas gender, age, number of skin lesions, and presence of lymph nodes were not. |
|  |  | headache | Headaches | Nervous system disorders | 10 |  |  |  |
|  |  | Phlebitis | Vascular infections and inflammations | Vascular disorders | 6 |  |  |  |
|  |  | Injection site pain | Administration site reactions | General disorders and administration site conditions | 4 |  |  |  |
|  |  | Malaise | General system disorders NEC | General disorders and administration site conditions | 4 |  |  |  |
|  |  | Insomnia | Sleep disorders and disturbances | Psychiatric disorders | 4 |  |  |  |
|  |  | Bradycardia | Cardiac arrhythmias | Cardiac disorders | 3 |  |  |  |
|  |  | Fever | Body temperature conditions | General disorders and administration site conditions | 3 |  |  |  |
|  |  | Vertigo | Inner ear and VIIIth cranial nerve disorders | Ear and labyrinth disorders | 1 |  |  |  |
|  |  | Weight decreased | Physical examination and organ system status topics | Investigations | 1 |  |  |  |
|  |  | Nausea | Gastrointestinal signs and symptoms | Gastrointestinal disorders | 1 |  |  |  |
|  |  | Vomiting | Gastrointestinal signs and symptoms | Gastrointestinal disorders | 1 |  |  |  |
|  |  | Pruritus | Epidermal and dermal conditions | Skin and subcutaneous tissue disorders | 1 |  |  |  |
|  |  | Decreased appetite / anorexia | Appetite and general nutritional disorders | Metabolism and nutrition disorders | 1 |  |  |  |
| 2005, Name | Sb^v^ (148) | Electrocardiogram abnormal | Cardiac and vascular investigations (excl enzyme tests) | Investigations | 69 | NR |  | Not reported separately for ML (except for Electrocardiographical alterations).The most encountered alteration in blood count was eosinophilia (17.5%). Blood biochemistry showed an increase in transaminases activity, of 9.3 % in TGO and 6.8% in TGP. In uranalysis (UA), the main alteration was pyuria (12.8%). Electrocardiographic alterations were found in 20.3% of the patients that used the 20mg Sb^v^/kg/day for 20 days schedule and in 46.6% of those who usedthe 30-day schedule. Sixty-two patients (17%) had already been treated with pentavalent antimony. Of these, only 42% (26 patients) had completed treatment. The found laboratory alterations are compatible with the literature, with the exception of the most frequent finding here described, namely, eosinophilia, more restrictly divulged. Electrocardiographic alterations occurred more often in the 30-day regimen (148 ML patients). This difference was statistically significant (compared to the LC 20-day regimen). This datum corroborates the observations that more prolonged therapeutic schedules tend to cause more side effects. |
| 2004, Calvopina | Itraconazole (13) | NR | NR | NR | NR | NR |  | No side-effects (clinical or laboratory) related to itraconazole were reported during treatment, and no subject abandoned treatment. |
| 2000, Oliveira-Neto | MA-LD (35)* | Arthralgia | Joint disorders | Musculoskeletal and connective tissue disorders | 16 | NR |  | Side effects were noted in 25 of the 35 patients (71.4%), including patients treated with regimens other than MA-LD, for 30 days (MA-LD 5 mg/kg/day for 40 days and 15 mg/kg/day for 10 days). These side effects were mild in the majority of patients, being more intense in the four patients who received the schedule of 15 mg/kg/day during 10-days. The most common side-effects noted were: arthralgias (16 patients – 45.7%), astenia (13 patients – 37.1%), myalgias (10 patients – 27.7%), nausea and vomiting (7 patients – 20%), alterations in electrocardiogram – increased QT interval, arrhythmia, extrasystoles, diffuse alterations in repolarization (7 patients – 20%), hematological abnormalities – eosinophilia, leukopenia, decreased hematocrit – (6 patients – 17.1%), local pain at the site of the injection (6 patients – 17.1%). In five patients (14.2%) worsening of lesions was noted at the first injections. Four patients (11.4%) presented fever at the beginning of therapy. Two cases developed herpes zoster and three presented herpes simplex by the end of therapy. Elevation of aminotransferase levels was noted in two patients and raised amylase and lipase in one patient. These three patients received the dose of 15 mg/kg/day during 10-days. |
|  |  | Asthenia | General system disorders NEC | General disorders and administration site conditions | 13 |  |  |  |
|  |  | Myalgia | Muscle disorders | Musculoskeletal and connective tissue disorders | 10 |  |  |  |
|  |  | Nausea and vomiting | Gastrointestinal signs and symptoms | Gastrointestinal disorders | 7 |  |  |  |
|  |  | Electrocardiogram abnormal | Cardiac and vascular investigations (excl enzyme tests) | Investigations | 7 |  |  |  |
|  |  | Blood disorder | Hematological disorders NEC | Blood and lymphatic system disorders | 6 |  |  |  |
|  |  | Injection site pain | Administration site reactions | General disorders and administration site conditions | 6 |  |  |  |
|  |  | Fever | Body temperature conditions | General disorders and administration site conditions | 4 |  |  |  |
|  |  | Herpes zoster | Viral infectious disorders | Infections and infestations [10021881] | 2 |  |  |  |
|  |  | Herpes simplex | Viral infectious disorders | Infections and infestations | 3 |  |  |  |
| 1997, Llanos-Cuentas | SSG (41) | Headache | Headaches | Nervous system disorders | 32 | NR The text implies that EA losses were caused according to severity. | 2 (thrombocytopenia) | The rates of clinical AE were similar among both groups, but there was a trend for greater severity in SSG + allopurinol group. Severe adverse events caused the withdrawal of 11 patients (3 in the SSG group and 8 in the SSG + allopurinol group). Eight patients withdrew because of severe thrombocytopenia (2 from the SSG group and 6 from SSG + allopurinol group), 1 patient developed severe bradycardia, 1 patient from SSG + allopurinol group stopped treatment because of hepatic abnormality and 1 withdrew because of a new illness (typhoid fever). The more frequent symptoms were headache (81.5%), arthralgia (75.3%), myalgia (67.9%), chills (42%), fever (39.5%), abdominal pain (33.3%), and anorexia (25.9%). Three patients developed ophthalmic herpes zoster (1 in the SSG group and 2 in the SSG + allopurinol group) by the end of treatment. The most frequent laboratory AE was hematologic abnormality (anemia, leukopenia and thrombocytopenia). Hepatic abnormality was manifested mainly as an increase in the AST or ALT level, usually less than two times the baseline value. The rates of hepatic AE was similar among both groups. No significant changes in serum creatinine levels were found. Minor and transient electrocardiographic abnormalities were detected during treatment of 89% of the patients, and no difference was detected between groups. |
|  |  | Arthralgia | Joint disorders | Musculoskeletal and connective tissue disorders | 32 |  |  |  |
|  |  | Myalgia | Muscle disorders | Musculoskeletal and connective tissue disorders | 31 |  |  |  |
|  |  | Chills | General system disorders NEC | General disorders and administration site conditions | 15 |  |  |  |
|  |  | Fever | Body temperature conditions | General disorders and administration site conditions | 15 |  |  |  |
|  |  | Abdominal pain | Gastrointestinal signs and symptoms | Gastrointestinal disorders | 14 |  |  |  |
|  |  | Decreased appetite / anorexia | Appetite and general nutritional disorders | Metabolism and nutrition disorders | 9 |  |  |  |
|  |  | Diarrhoea | Gastrointestinal motility and defaecation conditions | Gastrointestinal disorders | 6 |  |  |  |
|  |  | Paraesthesia | Neurological disorders NEC | Nervous system disorders | 4 |  |  |  |
|  |  | Vomiting | Gastrointestinal signs and symptoms | Gastrointestinal disorders | 3 |  |  |  |
|  |  | Epistaxis | Upper respiratory tract disorders (excl infections) | Respiratory, thoracic and mediastinal disorders | 2 |  |  |  |
|  |  | Herpes zoster | Viral infectious disorders | Infections and infestations | 1 |  |  |  |
|  |  | Gingival bleeding | Dental and gingival conditions | Gastrointestinal disorders | 0 |  |  |  |
|  |  | Blood disorder | Hematological disorders NEC | Blood and lymphatic system disorders | 11 |  |  |  |
|  |  | Hepatic enzyme abnormal | Hepatobiliary investigations | Investigations | 6 |  |  |  |
|  |  | Renal function test abnormal | Renal and urinary tract investigations and urinalyses | Investigations | 9 |  |  |  |
|  | SSG + allopurinol (40) | Headache | Headaches | Nervous system disorders | 34 |  | 9 (6 thrombocytopenia, 1 bradicardia, 1 hepatic abnormality and 1 new illness) |  |
|  |  | Arthralgia | Joint disorders | Musculoskeletal and connective tissue disorders | 29 |  |  |  |
|  |  | Myalgia | Muscle disorders | Musculoskeletal and connective tissue disorders | 24 |  |  |  |
|  |  | Chills | General system disorders NEC | General disorders and administration site conditions | 19 |  |  |  |
|  |  | Fever | Body temperature conditions | General disorders and administration site conditions | 17 |  |  |  |
|  |  | Abdominal pain | Gastrointestinal signs and symptoms | Gastrointestinal disorders | 13 |  |  |  |
|  |  | Decreased appetite / anorexia | Appetite and general nutritional disorders | Metabolism and nutrition disorders | 12 |  |  |  |
|  |  | Diarrhoea | Gastrointestinal motility and defaecation conditions | Gastrointestinal disorders | 4 |  |  |  |
|  |  | Paraesthesia | Neurological disorders NEC | Nervous system disorders | 4 |  |  |  |
|  |  | Vomiting | Gastrointestinal signs and symptoms | Gastrointestinal disorders | 3 |  |  |  |
|  |  | Epistaxis | Upper respiratory tract disorders (excl infections) | Respiratory, thoracic and mediastinal disorders | 3 |  |  |  |
|  |  | Herpes zoster | Viral infectious disorders | Infections and infestations | 2 |  |  |  |
|  |  | Gingival bleeding | Dental and gingival conditions | Gastrointestinal disorders | 2 |  |  |  |
|  |  | Blood disorder | Haematological disorders NEC | Blood and lymphatic system disorders | 12 |  |  |  |
|  |  | Hepatic enzyme abnormal | Hepatobiliary investigations | Investigations | 5 |  |  |  |
|  |  | Renal function test abnormal | Renal and urinary tract investigations and urinalyses | Investigations | 8 |  |  |  |
| 1996, Romero | AS (21)* | Azotaemia | Renal disorders (excl nephropathies) | Renal and urinary disorders | 1 | NR |  | Not reported separately for the study arm. In the total population (21), one patient had azotemia on the day 20 of the treatment that remitted 4 days later. Five patients had mild proteinuria that regressed after treatment. Two patients underwent audiometric tests; one presented on the 20th of treatment, hearing loss for the sounds of high frequency and the changes persisted in the control audiometry 3 months later; at the second patient the test was normal. None patient had signs or symptoms of toxicity clinically detectable vestibular or auditory. Seventeen (80.9%) patients complained of pain at the injection site in the evaluation performed on the tenth day of treatment, 18 (85.7%) on the twentieth day and 5 (23.8%) up to one month after the treatment. |
|  |  | Proteinuria | Urinary tract signs and symptoms | Renal and urinary disorders | 5 |  |  |  |
|  |  | Deafness | Hearing disorders | Ear and labyrinth disorders | 1 |  | 1 (hearing loss for the sounds of high frequency) |  |
|  |  | Injection site pain | Administration site reactions | General disorders and administration site conditions | 18 |  |  |  |
| 1995, Oliveira | MA (51) | NR | NR | NR | NR | NR |  | NR |
| 1994, Franke | SSG, 28 days (20) | Arthralgia | Joint disorders | Musculoskeletal and connective tissue disorders | 10 | Treatment suspended due to AE | 2 (thrombocytopenia) | Although more P40 patients than P28 patients complained of arthralgias and myalgias. most complaints began before day 28.  Treatment was suspended prior to the end of therapy in two P28 patients and one P40 patient due to thrombocytopenia. Treatment was suspended for two days in one patient due to an abnormally high potassium level measured on day 7. Another sample of the patient's blood was taken on day 8 and all blood chemistry results were normal: treatment was resumed on day 9 and this patient received the full 40-day course of therapy. |
|  |  | Myalgia | Muscle disorders | Musculoskeletal and connective tissue disorders | 11 |  |  |  |
|  |  | Pruritus | Epidermal and dermal conditions | Skin and subcutaneous tissue disorders | 1 |  |  |  |
|  |  | Rash | Epidermal and dermal conditions | Skin and subcutaneous tissue disorders | 2 |  |  |  |
|  |  | Nausea | Gastrointestinal signs and symptoms | Gastrointestinal disorders | 6 |  |  |  |
|  |  | Decreased appetite / anorexia | Appetite and general nutritional disorders | Metabolism and nutrition disorders | 3 |  |  |  |
|  |  | Abdominal pain | Gastrointestinal signs and symptoms | Gastrointestinal disorders | 3 |  |  |  |
|  |  | Cough | Respiratory disorders NEC | Respiratory, thoracic and mediastinal disorders | 5 |  |  |  |
|  |  | Headache | Headaches | Nervous system disorders | 7 |  |  |  |
|  | SSG, 40 days (20) | Arthralgia | Joint disorders | Musculoskeletal and connective tissue disorders | 18 |  | 1 (thrombocytopenia) |  |
|  |  | Myalgia | Muscle disorders | Musculoskeletal and connective tissue disorders | 19 |  |  |  |
|  |  | Pruritus | Epidermal and dermal conditions | Skin and subcutaneous tissue disorders | 4 |  |  |  |
|  |  | Rash | Epidermal and dermal conditions | Skin and subcutaneous tissue disorders | 4 |  |  |  |
|  |  | Nausea | Gastrointestinal signs and symptoms | Gastrointestinal disorders | 6 |  |  |  |
|  |  | Decreased appetite / anorexia | Appetite and general nutritional disorders | Metabolism and nutrition disorders | 5 |  |  |  |
|  |  | Abdominal pain | Gastrointestinal signs and symptoms | Gastrointestinal disorders | 4 |  |  |  |
|  |  | Cough | Respiratory disorders NEC | Respiratory, thoracic and mediastinal disorders | 4 |  |  |  |
|  |  | Headache | Headaches | Nervous system disorders | 3 |  |  |  |
| 1993, Zocoli | ketoconazole (25) Sb^v^ (98) Sb^v^ + sulfa (180) | NR | NR | NR | NR | NR |  | NR |
| 1991, Kopke | MA (43)* | Eosinophilia | White blood cell disorders | Blood and lymphatic system disorders | 13 | Treatment suspended due to AE |  | Not reported separately for each study arm. Consider study with CL (26 patients) and MCL (17 patients), with total of 43 patients.The drug showed low toxicity and was well tolerated. However, the authors found a small increase in the levels of hepatic enzymes, eosinophilia and leukopenia with neutropenia (causing treatment interruption and withdrawal from the study of two patients). Electrocardiographic changes mainly in the ventricular repolarization were insignificant and reversible as the drug was stopped at the end of the treatment. Side effects like myalgia, nausea, vomit, inappetence, trembling, diarrhea, fever and urticaria were slight and not responsible for the interruption of therapy, except for one case of acute respiratory insufficiency (Jarisch-Herxheimer reaction). |
|  |  | Leukopenia | White blood cell disorders | Blood and lymphatic system disorders | 5 |  | 2 |  |
|  |  | Red blood cell sedimentation rate decreased | Haematology investigations (incl blood groups) | Investigations | 14 |  |  |  |
|  |  | Hepatic enzyme increased | Hepatobiliary investigations | Investigations | 16 |  |  |  |
|  |  | Electrocardiogram abnormal | Cardiac and vascular investigations (excl enzyme tests) | Investigations | 11 |  |  |  |
|  |  | Jarisch-Herxheimer reaction | Immune disorders NEC | Immune system disorders | 4 |  | 1 |  |
| 1991, Saenz | SSG (16) | Arthralgia | Joint disorders | Musculoskeletal and connective tissue disorders | 10 | Treatment suspended due to AE |  | Subjective musculoskeletel complaints were common in this study: 10 of 16 patients (62%) had arthralgias and nine (56%) had myalgias. Abdominal complaints were less frequent in that only 2 patients had either nausea, abdominal pain, diarrhea, or anorexia. Six of 16 patients (37%) had abnormalities of liver function tests signified by elevations of both SGOT and SGPT. There were three patients in whom therapy was prematurely terminated because of high liver function tests. |
|  |  | Myalgia | Muscle disorders | Musculoskeletal and connective tissue disorders | 9 |  |  |  |
|  |  | Abdominal pain, including nausea, diarrhea or anorexia | Gastrointestinal signs and symptoms | Gastrointestinal disorders | 2 |  |  |  |
|  |  | Hepatic enzyme increased | Hepatobiliary investigations | Investigations | 6 |  | 3 |  |
|  |  | Electrocardiogram abnormal | Cardiac and vascular investigations (excl enzyme tests) | Investigations | 1 |  |  |  |
| 1990, Franke | SSG (29) | Myalgia | Muscle disorders | Musculoskeletal and connective tissue disorders | 24 | Treatment suspended due to AE |  | Side effects of this treatment regimen included T-wave inversion on electrocardiogram (4 patients), abnormal liver function tests (10 patients), and musculoskeletal pain (24 patients). No side effects occurred during week 1 of therapy.  Two patients discontinued treatment for AE (1 due to a low platelet level and 1 due to a high AST level). |
|  |  | Arthralgia | Joint disorders | Musculoskeletal and connective tissue disorders | 24 |  |  |  |
|  |  | Nausea | Gastrointestinal signs and symptoms | Gastrointestinal disorders | 8 |  |  |  |
|  |  | Decreased appetite / anorexia | Appetite and general nutritional disorders | Metabolism and nutrition disorders | 8 |  |  |  |
|  |  | Abdominal pain | Gastrointestinal signs and symptoms | Gastrointestinal disorders | 8 |  |  |  |
|  |  | Headache | Headaches | Nervous system disorders | 6 |  |  |  |
|  |  | Herpes zoster | Viral infectious disorders | Infections and infestations | 1 |  |  |  |
|  |  | Electrocardiogram abnormal | Cardiac and vascular investigations (excl enzyme tests) | Investigations | 4 |  |  |  |
|  |  | Blood disorder | Haematological disorders NEC | Blood and lymphatic system disorders | 2 |  | 1 (low platelet level) |  |
|  |  | Hepatic enzyme increased | Hepatobiliary investigations | Investigations | 10 |  | 1 (high AST level) |  |
| 1989, Sampaio | MA (40)* | Hepatic enzyme increased | Hepatobiliary investigations | Investigations | 5 | Treatment suspended due to AE |  | Not reported separately for ML. The AE were evaluated only for some patients. Increases in transaminases or alkaline phosphatase were observed in 24% of patients. Electrokaryographic abnormalities were observed in 22% (repolarization disorders, sinus bradycardia or T wave alteration). However, the side effects observed exceptionally led to discontinuation of treatment. |
|  |  | Blood urea increased | Renal and urinary tract investigations and urinalyses | Investigations | 1 |  |  |  |
|  |  | Electrocardiogram abnormal | Cardiac and vascular investigations (excl enzyme tests) | Investigations | 8 |  |  |  |
|  | Sb^v^ (21)* | Hepatic enzyme increased | Hepatobiliary investigations | Investigations | 6 |  |  |  |
|  |  | Blood urea increased | Renal and urinary tract investigations and urinalyses | Investigations | 1 |  |  |  |
|  |  | Electrocardiogram abnormal | Cardiac and vascular investigations (excl enzyme tests) | Investigations | 4 |  |  |  |
|  | SSG (26)* | Hepatic enzyme increased | Hepatobiliary investigations | Investigations | 5 |  |  |  |
|  |  | Electrocardiogram abnormal | Cardiac and vascular investigations (excl enzyme tests) | Investigations | 2 |  |  |  |
| 1960, Sampaio | d-AMB (11) | Chills | General system disorders NEC | General disorders and administration site conditions | NR | NR |  | Chills and fever were controlled by previous administration of corticosteroids and dipyrone. Phlebitis was minimal. The level of blood urea increase, but returned to normal at the end of treatment. The only unusual complication is a case of a 20 week pregnant woman, who presented weakness of the lower limbs and could not stand up. Neurological examination showed only a motor deficit, which was attributed to hypocalcia and disappeared with calcium administration. She was discharged in obstetrical normal condition, but 15 days later had a premature fetus, which died after a few hours. |
|  |  | Fever | Body temperature conditions | General disorders and administration site conditions | NR |  |  |  |
|  |  | Phlebitis | Vascular infections and inflammations | Vascular disorders | NR |  |  |  |
|  |  | Foetal death | Abortions and stillbirth | Pregnancy, puerperium and perinatal conditions | NR |  |  |  |

**&**: AE were considered per treatment and not per patient. *****the number of participants reporting AE is different from the number of participants reporting a cure rate. **ABLC**: Amphotericin B, lipid complex. **AE:** Adverse Event. **AS**: Aminosidine sulphate. **c-AMB**: Amphotericin B colloidal dispersion. **CTCAE**: Common Terminology Criteria for Adverse Events. **DAIDS**: Division of AIDS Table for Grading of Severity of Adult and Pediatric Adverse Events. **d-AMB**: Deoxycholate amphotericin B. **HLGT**: High Level Group Terms. **L-AMB**: Lipossomal amphotericin B. **MA**: Meglumine antimonate. **MA-LD**: Meglumine antimonate low dose. **MF**: Miltefosine. **NEC**: not elsewhere classified. **NR**: not reported. **PENT**: pentamidine. **PT**: Preferred Term. **Sb^v^**: Antimonial pentavalent. **SOC**: System Organ Classes. **SSG**: Sodium stibogluconate.
