# Supplementary material for: The cure rate after different treatments for mucosal leishmaniasis in the Americas: A systematic review
Source: PLoS Negl Trop Dis. 2022 Nov 17;16(11):e0010931. doi: 10.1371/journal.pntd.0010931 (PMC9714886; doi:10.1371/journal.pntd.0010931)
Supplement: S4 Table — AE: Adverse Event. HLGT: High Level Group Terms. L-AMB: Liposomal amphotericin B. MF: Miltefosine. Sbv: Antimonial pentavalent. SOC: System Organ Classes. SSG: Sodium stibogluconate. (DOCX) [file pntd.0010931.s005.docx]

**S4 Table. High Level Group Terms for the investigation System Organ Classes term by therapy**

| **HLGT for Investigation SOC term by therapy** | **Number of AE reported** | **% AE by therapy** |
| --- | --- | --- |
| **Sb^v^** | **218** | **100.0%** |
| Cardiac and vascular investigations (excl enzyme tests) | 120 | 55.0% |
| Hepatobiliary investigations | 62 | 28.4% |
| Haematology investigations (incl blood groups) | 14 | 6.4% |
| Renal and urinary tract investigations and urinalyses | 12 | 5.5% |
| Gastrointestinal investigations | 9 | 4.1% |
| Physical examination and organ system status topics | 1 | 0.5% |
| **SSG + allopurinol** | **13** | **100.0%** |
| Renal and urinary tract investigations and urinalyses | 8 | 61.5% |
| Hepatobiliary investigations | 5 | 38.5% |
| **L-AMB** | **3** | **100.0%** |
| Renal and urinary tract investigations and urinalyses | 2 | 66.7% |
| Hepatobiliary investigations | 1 | 33.3% |
| **MF** | **2** | **100.0%** |
| Gastrointestinal investigations | 2 | 100.0% |
| **Fluconazole** | **1** | **100.0%** |
| Hepatobiliary investigations | 1 | 100.0% |
| **Total Geral** | **237** |  |

**AE:** Adverse Event. **HLGT**: High Level Group Terms. **L-AMB**: Lipossomal amphotericin B. **MF**: Miltefosine. **Sb^v^**: Antimonial pentavalent. **SOC**: System Organ Classes. **SSG**: Sodium stibogluconate.
