# Supplementary material for: The cure rate after different treatments for mucosal leishmaniasis in the Americas: A systematic review
Source: PLoS Negl Trop Dis. 2022 Nov 17;16(11):e0010931. doi: 10.1371/journal.pntd.0010931 (PMC9714886; doi:10.1371/journal.pntd.0010931)
Supplement: Attachment — Submitted filename: Response to Reviewers_oct.2022_GC.docx [file pntd.0010931.s006.docx]

October 14, 2022

Dear Gregory Deye and Ricardo Fujiwara

Academic and Section Editors

PloS Neglected Tropical Diseases

Ref: *"The cure rate after different treatments for mucosal leishmaniasis in the Americas: a systematic review*" (PNTD-D-22-00981)

Thank you for handling and considering our submission to *PloS Neglected Tropical Diseases*. We are also grateful to the reviewers for their time and valuable contributions to improve the manuscript.

We have carefully considered all reviewers concerns and did our best to improve the text accordingly. In all cases we’ve included in the manuscript the required explanations. The modifications implemented in this revised version are summarized below together with specific answers to the questions placed by the reviewers.

Please let us know in case any other improvement in the manuscript becomes necessary.

Sincerely yours,

Corresponding author:

Janaína de Pina Carvalho

Instituto René Rachou - Fundação Oswaldo Cruz – Fiocruz Minas

Avenida Augusto de Lima, 1715

30190-001, Belo Horizonte, MG, Brazil

Phone/fax: + 55 31 3349-7837 / 7853 - E-mail: [janaina.carvalho@fiocruz.br](about:blank)

**Answers to the reviewers**

**Reviewer 1**

|  | **Reviewer’s comments** | **Author’s answer** |
| --- | --- | --- |
| Line 144 | 1- The cure criteria used by the clinical trials are clear in Table 2, but I think they should be clearly expressed in the text, including noting that an improvement of more than 90% was included as a cure. Perhaps this should be addressed in Methods item Outcomes line 144. | The suggestion was accepted, and this information was incorporated into the text on page 7, line 152. |
| Lines 536-8 | “in general, for all interventions, the AEs identified reflect the known toxicity pattern already informed in the manufacturers' technical information and literature”. I think that a review article on adverse effects of drugs used in the Americas (doi: 10.1016/j.actatropica.2011.02.007) line 538 should be cited here. | The suggestion was accepted, and the reference was incorporated into the text on page 53, line 555. |
| Lines 71-72 | 3- Lines 71-72: [3,4] have also been implicated with mucosal involvement, in addition to L. infantum and L. major [5]…Here I think it could also be added to L.amazonensis. | The suggestion was accepted, and this information was incorporated into the text on page 4, line 71 |
| Checklist PRISMA – Item 20 | 4- In item 20 of the checklist of items to include when reporting a systematic review or meta-analysis of PRISMA, the realization of the forest plot is considered ideal. This was not done, we asked why and thought it was worth commenting on it. | Thank you for the opportunity to comment on this point. Note that a graphic gathering the results from studies addressing the same intervention, along with the overall results (the forest plot), is displayed for each therapeutic approach in figures 2 to 8.  For interventions that could be grouped together (e.g. different amphotericin formulations), we presented the summarized cure rate for each subgroup and the final cure rate for the whole group. |
| Figures 2, 3,4,5,6, 7,8,9 | 5- Figures 2, 3,4,5,6, 7,8,9 regarding cure rates show vertical lines, mostly with negative values (for exemple: -1.00; -0.50, etc.). I wonder if these lines would not be dispensable and if negative values for cure rates would not be inappropriately used (lines 323 -366). | The default for displaying the forest plot figure in the CMA software requires a symmetrical scale and zero at the mid-point. We agree that for a pooled cure rate a negative measure does not make sense, but this graphical presentation cannot be changed. |
| Figure 10 | 6- In Figure 10 (a and b) in Outcome or Casuality I think it would be better or more appropriate to put moderate risk instead of mean risk. | The suggestion was accepted, and Figure 10 was corrected, replacing “mean” for “moderate” on page 48. |
| Table 1 and 2 | 7- I had difficulty understanding that the numbers placed in the first cell of Tables 1 and 2 referred to the bibliographic references of the authors, I think it is appropriate to include the word References next to the item (Year, Author). I think it would also be appropriate to include the total number of patients in the same table to make it clearer. | The suggestion was accepted, and the word “reference” was incorporated into the Tables 1, 2 and 3 (pages 12, 23 and 34)  The total number of patients treated is reported in the second column, in parentheses, and indicated in the legend as “N”. |
| Conclusions | Public health relevance could be more reinforced. | The suggestion was accepted, and this information was incorporated into the text on page 53-54, line 563-568 and 581. |

**Reviewer 2**

|  | **Reviewer’s comments** | **Author’s answer** |
| --- | --- | --- |
| Discussion | The conclusion is supported by the data under analysis and a very careful list of limitations with their respective explanations were included. In fact, the paragraphs dedicated to describe the study limitations can be very useful for the potential readers not familiarized with the peculiarities of mucocutaneous leishmaniasis in the Americas. Although authors mention something for the next steps in the field of treatment of ML, I feel that more emphasis should be dedicated to the possibility of improving the clinical and therapeutic data registries in the endemic countries in order to have the routine surveillance systems as a source of useful information regarding the challenge of achieving effective treatments. Also, it could be highlighted the urgent need to turn viable the conduction of an adequate powered RCT for comparing the current therapeutic options in a large pragmatic clinical trial involving all the ML cases treated in the Americas. | The suggestion was accepted, and this information was incorporated into the text on page 53-54, line 563-568 and 581. |
